# Soft and flexible poly(ethylene glycol) nanotubes for local drug delivery†
†Electronic supplementary information (ESI) available. See DOI: 10.1039/c8nr00603b


**DOI:** 10.1039/c8nr00603b

**Published:** 2018-05-01

**Authors:** B. Newland, C. Taplan, D. Pette, J. Friedrichs, M. Steinhart, W. Wang, B. Voit, F. P. Seib, C. Werner

**Affiliations:** a Leibniz-Institut für Polymerforschung Dresden , Max Bergmann Center of Biomaterials Dresden , Hohe Straße 6 , D-01069 Dresden , Germany . Email: newlandb@cardiff.ac.uk; b Brain Repair Group , School of Biosciences , Cardiff University , Cardiff , CF10 3AX , UK; c Technische Universität Dresden , Organic Chemistry of Polymers , 01062 Dresden , Germany; d Institut für Chemie neuer Materialien , Universität Osnabrück , Barbarastraße 7 , 49069 Osnabrück , Germany; e Charles Institute for Dermatology , University College Dublin , Dublin , Ireland; f Leibniz-Institut für Polymerforschung Dresden e.V. , Hohe Straße 6 , D-01069 Dresden , Germany; g Strathclyde Institute of Pharmacy and Biomedical Sciences , University of Strathclyde , 161 Cathedral Street , Glasgow , G4 0RE , UK

## Abstract

Nanotubes are emerging as promising materials for healthcare applications but the selection of clinically relevant starting materials for their synthesis remains largely unexplored. Here we present, for the first time, the synthesis of poly(ethylene glycol) (PEG) based nanotubes *via* the photopolymerization of poly(ethylene glycol) diacrylate and other diacrylate derivatives within the pores of anodized aluminum oxide templates. Template-assisted synthesis allowed the manufacture of a diverse set of polymeric nanotubes with tunable physical characteristics including diameter (∼200–400 nm) and stiffness (405–902 kPa). PEG nanotubes were subjected to cytotoxicty assessment in cell lines and primary stem cells and showed excellent cytocompatability (IC_50_ > 120 μg ml^–1^). Nanotubes were readily drug loaded but released the majority of the drug over 5 days. Direct administration of drug loaded nanotubes to human orthotopic breast tumors substantially reduced tumor growth and metastasis and outperformed i.v. administration at the equivalent dose. Overall, this nanotube templating platform is emerging as a facile route for the manufacture of poly(ethylene glycol) nanotubes.

## Abstract 2

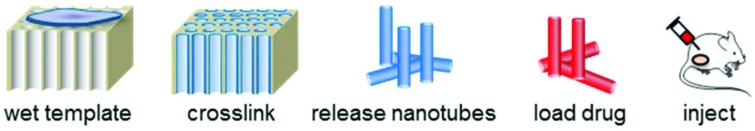
Soft/flexible PEG-based polymer nanotubes released doxorubicin over a sustained period and reduced tumor growth in a metastatic breast cancer model.

## 

Hollow high aspect ratio materials such as nanotubes are an interesting proposition for sustained drug release due to their combination of a high surface area to volume (for drug adsorption/loading) and an internal pore (for filling with drug).^1^ Carbon nanotubes have been heavily investigated for applications in gene and drug delivery.^2^–^4^ In their pristine form, without wall functionalization, carbon nanotubes can be loaded with certain drug molecules such as doxorubicin *via* π-stacking.^2^ However, a range of functionalization strategies can be pursued to facilitate the loading of specific molecules. Carbon nanotubes are particularly interesting for intracellular drug delivery because they have been shown to be taken up by multiple cell types *via* energy-independent mechanisms regardless of the functionalization strategy used.^5^ However, this intrinsic ability to pierce the cell membrane can be associated with cellular toxicity.^6^ Whilst the toxicity of carbon nanotubes is multifactorial (length, rigidity, impurities *etc*.^7^), they are rarely used in their pristine state as they require functionalization to improve their biocompatibility and stability (as a dispersed suspension). Poly(ethylene glycol) (PEG) or diamino-triethylene glycol polymers are commonly used for these purposes.^8^

Our hypothesis was to negate the need for a carbon nanotube starting material, instead using PEG as the nanotube bulk material thus eliminating the need for post modifications. As PEG can be used to improve the biocompatibility of nanoparticles,^9^ we speculated that non-toxic nanotubes could be produced. Porous anodized aluminum oxide (AAO) has served as a template for creating nanomaterials composed of silica,^10^ carbon,^11^ DNA,^12^ proteins,^13^ and polystyrene^14^ (reviewed elsewhere^15^,^16^). Herein, for the first time, we photopolymerize poly(ethylene glycol) diacrylate (PEGDA) and other diacrylate monomers within the pores of anodized aluminium oxide templates to create polymer nanotubes. Three different polymer nanotubes (herein termed PEG, Phos and Bisphenol) were successfully synthesized *via* the photopolymerization of three different divinyl monomers: poly(ethylene glycol) diacrylate (PEGDA), bis[2-(methacryloyloxy)ethyl] phosphate and bisphenol A ethoxylate diacrylate respectively. Precursor solutions containing the divinyl monomer and 2-hydroxy-2-methylpropiophenone as a photoinitiator were prepared in acetone and added to porous anodized aluminum oxide (AAO) membranes (Scheme 1). Fig. 1 shows the porous structure of the anodized aluminum oxide template prior to filling with the precursor solution (average pore diameter of the anodized aluminum oxide template was 224 nm ± 44 nm (Fig. S1†)).

**Scheme 1:**
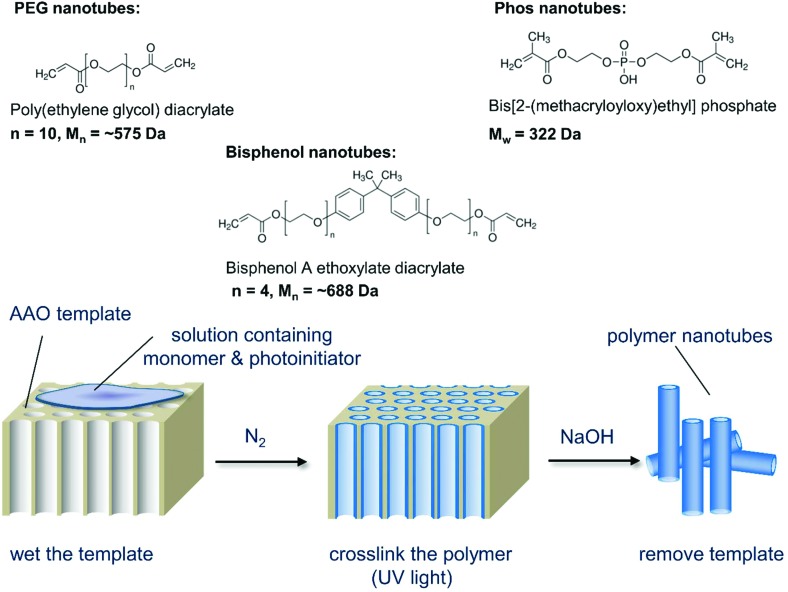
Schematic depiction of the nanotube synthesis process showing the three monomers used and the simple three-step procedure that can be used to create a variety of polymer nanotubes.

**Fig. 1:**
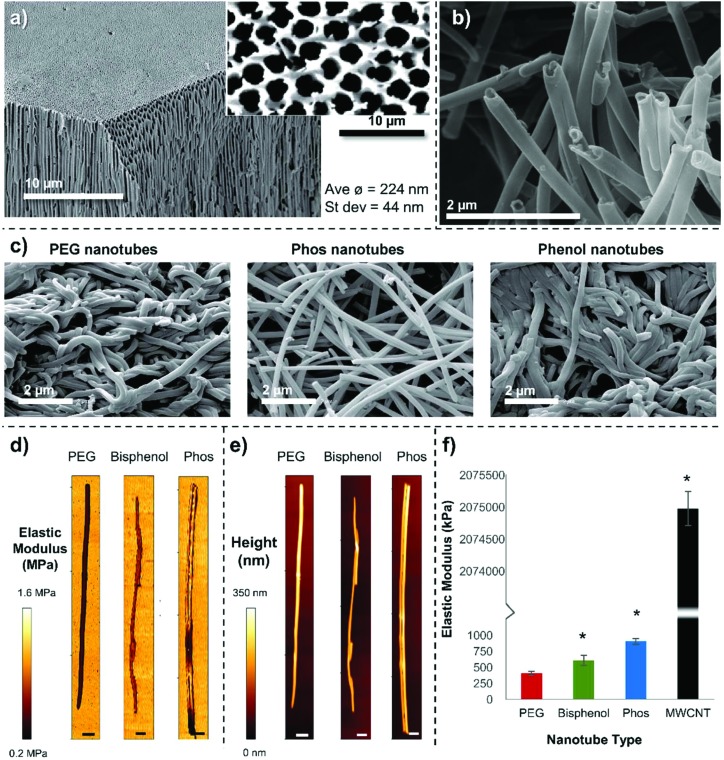
Nanotube characterization (a) SEM image of a anodized aluminum oxide membrane cut through and tilted to show the upper surface (high magnification and non-tilted image shown inset) and pore structure extending through the membrane. (b) A higher magnification image of PEG nanotubes, and (c) lower magnification images of the three different polymer nanotubes showing how they are orientated differently once dry. (d) Representative AFM force maps, with (e) corresponding height maps (scale bars = 100 nm). (f) A split axis graph showing that the Phos nanotubes are stiffer than the PEG and Bisphenol nanotubes but are ∼2000 times less stiff than MWCNTs (*n* = 3, error bars represent ± standard deviation, * show statistical significant difference from PEG nanotubes (*P* ≤ 0.05, one way ANOVA)).

The monomers were successfully added to the template, which formed a continuous inner layer on the template pore wall. They were then polymerized into a crosslinked tube upon irradiation by UV light and released *via* template dissolution (30 minutes in sodium hydroxide^17^,^18^) (Raman spectroscopy shown in Fig. S2†). Partial dissolution of the template (3 minutes in sodium hydroxide) was also performed to visualize the nanotubes whilst still in the template (Fig. S3†). These images show the difference between the upper and lower surface of the templates indicating that the pore structure is not constant throughout the template. For this reason, the monomer solution was added to both sides of the template during synthesis. Representative SEM images of PEG, Phos and Bisphenol polymer nanotubes are shown in Fig. 1. MWCNTs with a diameter close that of the polymer nanotubes were used in these studies as a comparison material (SEM image of the MWCNTs is shown in Fig. S4†).

The PEG nanotubes are approximately double the length of their carbon nanotube counterparts (average length of PEG nanotubes was 15.4 μm (±11.4 μm) *vs.* 6.2 μm (±3.0 μm) for MWCNT) with some PEG nanotubes reaching 60 μm in length (Fig. S5†). All three polymer nanotube types had a smaller distribution of diameters than MWCNT (Fig. S6†) with the nanotube diameter being dictated by the pore diameter of the anodized aluminum template (average diameter or PEG nanotubes was 202 nm). MWCNTs showed a large range in diameter ranging from 60 nm to 800 nm with an average diameter of 164 nm (manufacturer's diameter range given as 110 nm–170 nm). Transmission electron microscope (TEM) images of the four different nanotube types are shown in Fig. S7† with additional higher magnification images of the nanotube ends. These images also show the regular diameter of the polymer nanotubes in comparison to the wide variation displayed by MWCNTs.

Proof of concept experiments showed that we can synthesize PEG nanotubes of two different diameters (average 202 nm (±37 nm) and 402 nm (±57 nm)) (Fig. S8†) and different lengths (average 7.5 μm (±3.2 μm) and 30.9 μm (±19.0 μm)) (Fig. S9†) depending on the type of anodized aluminum oxide template used.

Representative SEM images (Fig. 1a–c) show that the different polymer nanotubes in their dry state appear to have different morphologies, with PEG and Bisphenol nanotubes being very entangled around each other, in contrast to the Phos nanotubes that are less curved and not so intertwined; this suggests that the Phos nanotubes are less flexible than the others.

We reasoned that this difference in structure may be due to the shorter chain length of the bis[2-(methacryloyloxy)ethyl] phosphate monomer used to make the Phos nanotubes (*M*_w_ = 322 Da, 2 PEG units), than the PEGDA monomer used to make the PEG nanotubes (*M*_w_ ∼ 575 Da, 10 PEG units). A shorter monomer chain length would result in a higher crosslinking density and therefore a more rigid structure. To analyze the mechanical properties of the nanotubes, atomic force microscopy was performed to create force maps, from which the elastic modulus (Young's modulus) was determined (Fig. 1d–f). Indentation of the nanotube wall, showed that PEG nanotubes (405 kPa, ±32) were significantly softer than Phos nanotubes (902 kPa, ±42), with Bisphenol nanotubes in between (607 kPa, ±79). This data suggests that the choice of monomer selected to make the nanotubes will not only affect its chemical composition, but also the mechanical properties of the polymer nanotubes. MWCNTs were ∼2000 times stiffer (Young's modulus of 2.07 GPa, ±0.36) showing that the polymer nanotubes are indeed very soft and flexible.

As an initial screen, to analyze the inherent cytotoxicity of the polymer nanotubes in direct comparison to MWCNTs, cells grown in culture were incubated with nanotubes ranging in concentration from 0 to 120 μg mL^–1^ for 1 or 3 days. Mouse fibroblasts (3T3) (Fig. S10†), human mesenchymal stem cells (MSCs) (Fig. S11†) and human breast epithelial cells (MCF-10A) (Fig. S12†) were used. No decrease in 3T3 cell viability occurred for all nanotube types at any of the concentrations tested (Fig. S10a†). Images show no change in cell morphologies, although the MWCNTs appeared to be aggregated with the cells (Fig. S10b†). MSCs were the most affected by the nanotubes with a decrease in viability to 77% (Phos), 67% (Bisphenol) and 30% (MWCNT) after three days of incubation at 120 μg mL^–1^ (Fig. S11†). In these stem cell cultures Phos nanotubes and MWCNT aggregated at the cell surface. PEG nanotubes formed an even covering over the cells and well plate bottom and did not reduce stem cell viability. In a similar manner to the 3T3 cells, incubation of MCF-10A cells with nanotubes did not affect their viability for all nanotube types and concentrations tested (Fig. S12†). The next step was to assess whether these nanotubes could be used as a local drug delivery system.

Systemic delivery of anticancer drugs as part of an adjuvant chemotherapy regime for breast cancer patients is associated with severe adverse side effects. These effects can be classified into short term effects such as nausea/emesis, thromboembolism, stomatitis *etc*., and long term effects such as premature menopause/infertility, neuropathy and cardiac dysfunction.^19^ Doxorubicin is commonly used as part of the treatment regime for both early stage and metastatic breast cancer, but has severe toxicity issues.^20^ Local drug delivery strategies represent a way to enhance the effectiveness of the drug, whilst reducing its side effects, by releasing a high concentration of the drug focally to the tumor. Local drug delivery is already in use for a range of malignancies, and drug delivery devices in development take on a range of different forms including wafers, films, nanoparticles, gels and rods (reviewed elsewhere^21^). Because tumor resection is common in breast cancer one could envisage the application of a local drug delivery system at the surgical site. Such a drug delivery system would ideally have some of the following characteristics: (a) be injectable (ease of application), (b) release the drug over an extended period of time, (c) remain at the injection site (reduce off-target effects), (d) be inert and non-toxic, and (e) be biodegradable. Such a system could also potentially be used for preoperative drug delivery to the tumor site or, if proven effective enough, used instead of systemic drug administration.^22^

To assess whether these nanotubes would uptake the chemotherapeutic drug doxorubicin, we incubated the nanotubes in an aqueous solution of doxorubicin (100 μg mL^–1^, drug to nanotube ratio of 1 : 10 – see ESI† for details) for 24 hours. Qualitative and quantitative assessment showed that PEG and Phos nanotubes efficiently took up the doxorubicin (Fig. S14†) (PEG 99.6%, Phos 96.3%, Bisphenol 30.6% and MWCNT 30.0%). We had speculated that the Bisphenol nanotubes may be a good candidate for loading doxorubicin either *via* hydrophobic interactions or *via* π-stacking of electrons between the six-membered rings on both the nanotubes and the doxorubicin molecule; however, less doxorubicin was loaded to the Bisphenol nanotubes. Instead, it is probable that the negative charge of the phosphate groups of the Phos nanotubes is a prominent driving force behind the efficient uptake of doxorubicin, *via* electrostatic interaction with the primary amine group on doxorubicin.

Although PEG should have no formal charge, the PEG nanotubes are perhaps successful in drug loading *via* the acquisition of a negative charge during the synthesis process. The 30 minutes treatment in sodium hydroxide (to dissolve the anodized aluminum oxide template) would cause hydrolysis of some of the PEG chains to from carboxylic acid groups on the nanotube surfaces (Fig. 2). Analysis of the degradation of the PEG nanotubes in sodium hydroxide shows that the nanotubes were completely broken down after 3 hours at room temperature (Fig. 2c). Zeta potential analysis of the PEG nanotubes showed a negative surface charge, though this was similar for all types of nanotubes (Fig. S15†) indicating that the mechanism of drug loading is complex and may not just be due to electrostatic interaction, but a combination of driving forces. Interestingly, the MWCNTs showed the highest rise in surface charge upon loading with doxorubicin, suggesting that the drug is surface adsorbed,^2^,^23^ whereas the polymer nanotubes maybe loading not only by surface adsorption but also within the pore. One would expect that the nanotubes might be biodegradable in physiological conditions *via* ester hydrolysis or ether oxidation. Long term studies with crosslinked PEGDA hydrogels, using polyethylene diacrylamide as controls, showed that ester hydrolysis is the likely mechanism of crosslinked PEGDA degradation after subcutaneous implantation in the rat.^24^ Furthermore, the secretome of frustrated phagocytes containing acid and hydroxyl radicals, could cause oxidative biodegradation.^25^,^26^ However, caution should be exercised before extrapolating analysis of PEGDA hydrogels to nanotubes formed from PEGDA due to differences in crosslink density and material size.

**Fig. 2:**
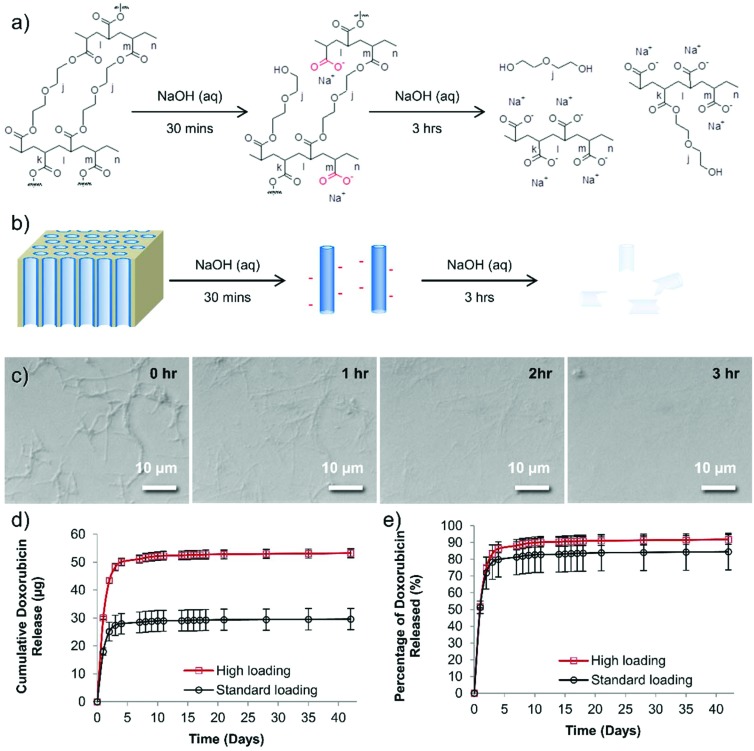
A proposed reaction scheme for the degradation of PEG nanotubes. (a) Ester hydrolysis of crosslinked PEGDA to yield carboxylic acid groups. (b) A schematic diagram showing the initial change in nanotube charge upon release from the template, and subsequent destruction of the nanotubes if left in sodium hydroxide. (c) Time lapse light microscopy of the degradation of PEG nanotubes left in 1 molar sodium hydroxide for 3 hours. (d) Doxorubicin loaded PEG nanotubes release the drug for 42 days *in vitro*, (“standard” = 400 μg mL^–1^, “high” = 800 μg mL^–1^ of doxorubicin). (e) Approximately 80–90% of the doxorubicin is released in the first seven days, then only a small amount (1.2 μg for standard group or 2.1 μg for high group) was released over the following 35 days (*n* = 4, error bars represent ± the cumulative standard deviation).

Based on the cytotoxicity and loading data only PEG nanotubes were examined further while MWCNTs served as a reference. In order to assess the maximum loading of doxorubicin to PEG nanotubes, we first defined a dose of doxorubicin that would be used for *in vivo* studies. In line with previous work,^27^ 80 μg of doxorubicin per mouse (*i.e.* two bilateral tumors per mouse each treated with 40 μg) would be used for systemic administration, so this value served as the benchmark from which to determine what mass of nanotubes could uptake this amount of drug. Fig. S16† shows that a concentration of at least 4 mg mL^–1^ is required to fully load the nanotubes with doxorubicin, indicating that the maximum weight-to-weight ratio of doxorubicin to nanotube is 0.2 : 1. The release of doxorubicin from nanotubes loaded with either 40 μg of doxorubicin (standard dose used *in vivo* (henceforth termed “standard”) which matched the intravenous doxorubicin control dose) or 80 μg of doxorubicin (highest dose used *in vivo* henceforth termed “high”) was analyzed over a period of 42 days. The majority of the doxorubicin was released over the first seven days (28.4 μg for the “standard” group, 51.1 μg for the “high” group) (Fig. 2d and e). Then, after this period, a further 1.2 μg (“standard” group) or 2.1 μg (“high” group) was released. To provide context a recent study showed that even a doxorubicin dose as low as 0.4 μg mL^–1^ can be extremely toxic to prostate cancer cell lines grown *in vitro*,^28^ so although these are small amounts released they may still provide therapeutic benefit. Fig. 2e shows that even after this extended incubation time, not all the doxorubicin was released from the nanotubes (as readily visible from the red doxorubicin-associated color).

These data suggest that the PEG nanotubes not only draw doxorubicin out of solution more effectively than MWCNT but also release the drug over a 5-day period.

In order to assess the cytotoxicity of nanotubes in the presence and absence of loaded doxorubicin two human breast cancer cell lines were used: estrogen-responsive MCF-7 cells (Fig. S17†) and highly aggressive triple negative MDA-MB-231 cells (Fig. 3). MCF-7 cells are commonly used for analyzing drug delivery systems *in vitro*,^29^,^30^ and MDA-MB-231 cells is a good human metastatic breast cancer model;^27^,^31^–^33^ these were subsequently used here in an orthotopic breast cancer mouse model.^27^,^33^ MCF-7 cell viability was not affected by unloaded PEG nanotubes; however, doxorubicin loaded PEG nanotubes at 60 μg mL^–1^ reduced cell viability to 71% and 38% (Fig. S17†) after one day and three days, respectively. The doxorubicin loaded PEG nanotubes were directly compared to a positive control of freely diffusible doxorubicin at a concentration of 4 μg mL^–1^ which has previously been shown to be toxic to range of cell types.^28^

**Fig. 3:**
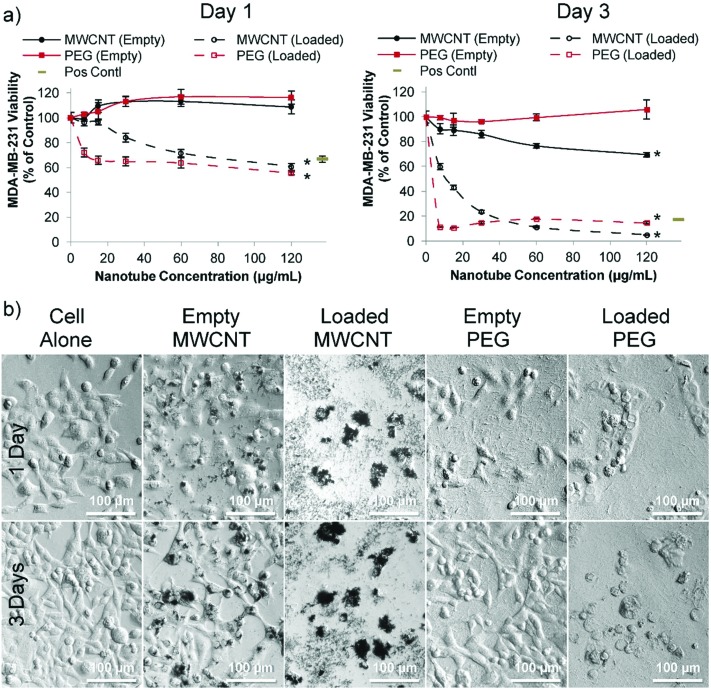
Cytotoxicity of doxorubicin loaded nanotubes with MDA-MB-231 breast cancer cells *in vitro*. (a) After one day (left hand graph), MDA-MB-231 cells incubated with either doxorubicin loaded MWCNTs or doxorubicin loaded PEG nanotubes show a significant decrease in viability which is further reduced by day three (as measured *via* the PrestoBlue assay). All nanotubes were subject to the same loading solutions (80 μg of doxorubicin per 1 mg of nanotubes (0.08 : 1 ratio)). Positive control is doxorubicin in solution at a concentration of 4 μg mL^–1^ (b) corresponding light microscope images of the 60 μg mL^–1^ nanotube concentration show the effect of the loaded nanotubes on cell morphology (*n* = 4), error bars represent ± standard deviation, * represents statistical significant difference to empty PEG nanotubes, (two way ANOVA with Tukey's multiple comparison test (*P* ≤ 0.05)).

For MDA-MB-231 cells, doxorubicin loaded PEG nanotubes at a concentration of 60 μg mL^–1^ reduced their viability to 64% and 18% after one and three days, respectively (Fig. 3a). Light microscopy analysis (Fig. 3b) suggested that both the empty and doxorubicin loaded MWCNTs clustered with the cells, which is in accordance with the theory that both positive and negatively charged MWCNTs are capable of cell penetration/uptake.^34^ Perhaps the large average diameter of the MWCNTs in this study limited their inherent toxicity in these cells.

In stark contrast to MWCNTs, for both the MCF-7 and MDA-MB-231 cell experiments, the PEG nanotubes formed an even layer over the well bottom, emphasizing that these inert nanotubes should function as a local sustained delivery device rather than performing direct cell penetration.

To prove the principle that that the PEG nanotubes could be used for local/sustained drug release we analyzed this system in an aggressive orthotopic metastatic breast cancer mouse model. The primary endpoint of this study was to assess clinical efficacy in mice treated with doxorubicin focally delivered *via* PEG nanotubes compared to an equivalent dose of intravenously administered doxorubicin.

MDA-MB-231 cells expressing the firefly luciferase gene allowed tumor growth to be monitored *via* non-invasive bioluminescence over time. The ability to image the implanted cells also allowed us to evaluate a secondary endpoint: whether treatment with the doxorubicin loaded nanotubes reduced the extent of metastases. Note that we also included in this study a “high” dose group where the nanotubes contained twice the amount of doxorubicin as the “standard” group (therefore also twice the amount as the intravenous doxorubicin group) (see Fig. 4a for experimental layout).

**Fig. 4:**
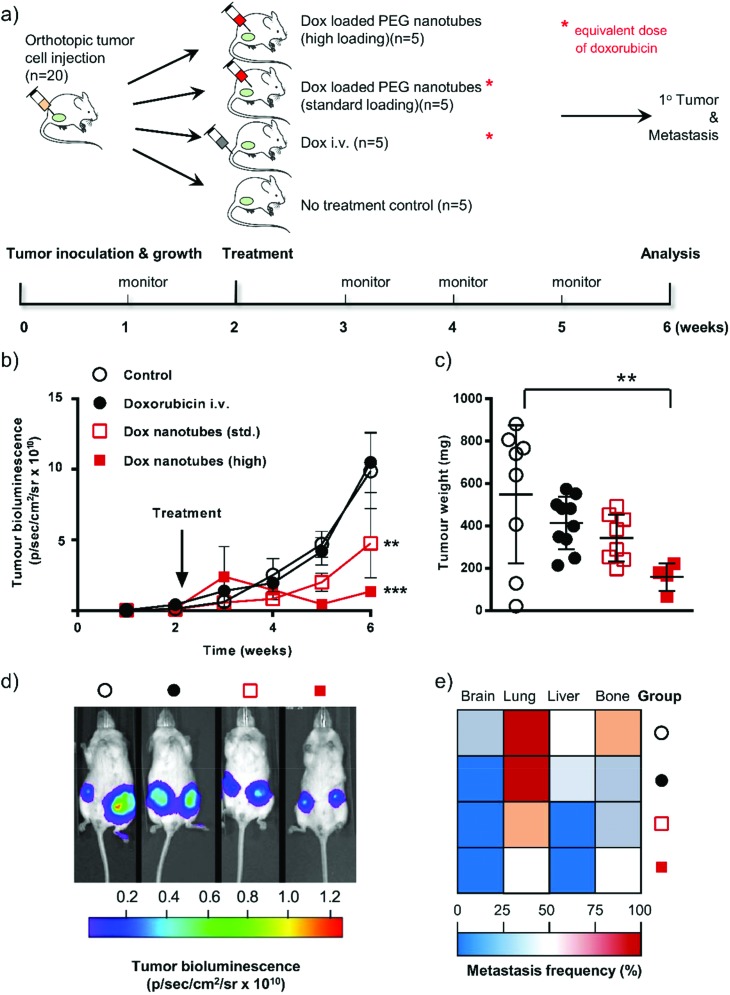
*In vivo* response of doxorubicin-loaded nanotubes in human orthotopic breast cancer. (a) Schematic diagram of the experimental approach showing the timeline and description of the groups. (b) Tumor growth in mice either untreated (open circle), 80 μg of doxorubicin by intravenous bolus injection (black circle), or doxorubicin-loaded nanotubes (standard dose 2 × 40 μg doxorubicin (open square) or high dose 2 × 80 μg of doxorubicin close to the bilateral orthotopic tumors). (c) Primary tumor weights were assessed at the end of the study (week 6). (d) Representative cancer cell-specific bioluminescence composite images at week 6. (e) Metastatic spread of cancer cells to organs at week 6. Statistical differences were determined using ANOVA (see materials and methods) **P ≤ 0.01, ***P ≤ 0.001; error bars represent ±SEM for tumor growth and ±SD tumor weights and are hidden in the plot-symbol when not visible, *n* = 4.

Tumor cell-associated bioluminescence signals of untreated and i.v. doxorubicin dosed animals were similar over the 6 weeks study period (Fig. 4b and d). The equivalent amount of doxorubicin (*i.e.* 40 μg per tumor) delivered locally *via* the PEG nanotubes showed reduced tumor growth compared to the i.v. doxorubicin group at week 5, which reached statistical significance by week 6 (*P* ≤ 0.01). Increasing the PEG nanotube doxorubicin loading to a dose of 80 μg per tumor (“high” dose) resulted in a more pronounced effect of the same trend (*P* ≤ 0.001 at week 6, when compared with the i.v. doxorubicin group). However, the “high” dose of doxorubicin delivered from the PEG nanotubes was likely outside the therapeutic window and induced toxicity. Therefore, three animals were euthanized due to ill health (7 and 13 days after initiation of therapy). We speculate that this could be due to cardiotoxicity, a well-known side effect of doxorubicin therapy.^20^,^35^ Assessment of tumor weight at the end of the study showed that i.v. doxorubicin dosing reduced the average tumor mass from 549 mg ± 326 (*i.e.* control) to 414 mg ± 124 (Fig. 4c). Local administration of the PEG nanotubes loaded with the “standard” doxorubicin dose substantially reduced the average tumor weight (343 mg ± 111). The “high” dose group significantly reduced the tumor burden (159 mg ± 64; *P* ≤ 0.01) compared to untreated controls (Fig. 4c). The aggressive nature of the MDA-MB-231 cancer model allowed us to analyze metastasis to the brain, lung, liver, and bone (evident in untreated control animals, Fig. 4e). Local treatment of primary breast tumors with nanotubes loaded with the “standard” 40 μg doxorubicin markedly reduced liver and lung metastases when compared to i.v. dosed animals.

These studies demonstrate that using PEG nanotubes to locally deliver doxorubicin to orthotopic breast tumors may be more effective in reducing tumor growth and metastasis. We selected doxorubicin because it is a clinical relevant cytotoxic agent which can be regarded as a benchmark therapeutic payload allowing direct comparison with both pre-clinical and clinically approved delivery systems.^36^ The physiochemical properties of doxorubicin are representative for many cytotoxic anticancer agents and thus doxorubicin serves as a useful indicator for other potential payloads. We note that sustained doxorubicin exposure has been linked to drug resistance.^37^ However, we speculate that our delivery system would also be applicable to other forms of cancer where local treatment is desirable, for example neuroblastoma and glioblastoma.^38^ Due to the versatile nature of the nanotube synthesis, and range of acrylate monomers commercially available, there are a myriad of ways that this platform technology can be built upon for further investigations.

## Conclusions

In summary, we show that poly(ethylene glycol) diacrylates as well as their phosphate and Bisphenol derivatives can be readily photopolymerized using anodized aluminum oxide templates to control the size of the resulting nanotubes. All synthesised nanotubes were flexible and approximately 2000 times softer than MWCNTs. The poly(ethylene glycol) nanotubes showed no toxicity in a panel of cell lines as well as primary stem cells over a broad concentration range (IC_50_ > 120 μg ml^–1^). The PEG nanotubes could be readily loaded with doxorubicin and subsequently release the drug for 42 days *in vitro* (longest time tested). PEG nanotube mediated focal delivery of doxorubicin to an orthotopic breast cancer mouse model showed greater reduction in tumor growth, and aa reduced metastasis rate, compared to a matching dose of doxorubicin administered intravenously. The simple synthesis procedure and the range of monomers available for nanotube synthesis means that a range of similar nanotubes can easily be synthesized for applications in focal drug delivery or beyond.

## Conflicts of interest

There are no conflicts to declare.

## Supplementary Material

Supplementary information

## References

[cit1] Hillebrenner H., Buyukserin F., Stewart J. D., Martin C. R. (2006). Nanomedicine.

[cit2] Liu Z., Sun X., Nakayama-Ratchford N., Dai H. (2007). ACS Nano.

[cit3] Pistone A., Iannazzo D., Ansari S., Milone C., Salamò M., Galvagno S., Cirmi S., Navarra M. (2016). Int. J. Pharm..

[cit4] Wen S., Liu H., Cai H., Shen M., Shi X. (2013). Adv. Healthcare Mater..

[cit5] Kostarelos K., Lacerda L., Pastorin G., Wu W., Wieckowski S., Luangsivilay J., Godefroy S., Pantarotto D., Briand J.-P., Muller S. (2007). Nat. Nanotechnol..

[cit6] Liu Y., Zhao Y., Sun B., Chen C. (2012). Acc. Chem. Res..

[cit7] Kostarelos K. (2008). Nat. Biotechnol..

[cit8] Battigelli A., Ménard-Moyon C., Da Ros T., Prato M., Bianco A. (2013). Adv. Drug Delivery Rev..

[cit9] Newland B., Zheng Y., Jin Y., Abu-Rub M., Cao H., Wang W., Pandit A. (2012). J. Am. Chem. Soc..

[cit10] Buyukserin F., Altuntas S., Aslim B. (2014). RSC Adv..

[cit11] Whitby M., Cagnon L., Thanou M., Quirke N. (2008). Nano Lett..

[cit12] Hou S., Wang J., Martin C. R. (2005). J. Am. Chem. Soc..

[cit13] Hou S., Wang J., Martin C. R. (2005). Nano Lett..

[cit14] Steinhart M., Wendorff J. H., Greiner A., Wehrspohn R. B., Nielsch K., Schilling J., Choi J., Gösele U. (2002). Science.

[cit15] Perry J. L., Martin C. R., Stewart J. D. (2011). Chem. – Eur. J..

[cit16] Karatas A., Hilal Algan A. (2017). Curr. Top. Med. Chem..

[cit17] Newland B., Thomas L., Zheng Y., Steinhart M., Werner C., Wang W. (2016). J. Interdiscip. Nanomed..

[cit18] Newland B., Leupelt D., Zheng Y., Thomas L. S. V., Werner C., Steinhart M., Wang W. (2015). Sci. Rep..

[cit19] Partridge A. H., Burstein H. J., Winer E. P. (2001). JNCI Monogr..

[cit20] Ansari L., Shiehzadeh F., Taherzadeh Z., Nikoofal-Sahlabadi S., Momtazi-borojeni A. A., Sahebkar A., Eslami S. (2017). Cancer Gene Ther..

[cit21] Wolinsky J. B., Colson Y. L., Grinstaff M. W. (2012). J. Controlled Release.

[cit22] Goldberg E. P., Hadba A. R., Almond B. A., Marotta J. S. (2002). J. Pharm. Pharmacol..

[cit23] Liu Z., Fan A. C., Rakhra K., Sherlock S., Goodwin A., Chen X., Yang Q., Felsher D. W., Dai H. (2009). Angew. Chem., Int. Ed..

[cit24] Browning M. B., Cereceres S. N., Luong P. T., Cosgriff-Hernandez E. M. (2014). J. Biomed. Mater. Res., Part A.

[cit25] Lynn A. D., Kyriakides T. R., Bryant S. J. (2010). J. Biomed. Mater. Res., Part A.

[cit26] Reid B., Gibson M., Singh A., Taube J., Furlong C., Murcia M., Elisseeff J. (2015). J. Tissue Eng. Regener. Med..

[cit27] Seib F. P., Tsurkan M., Freudenberg U., Kaplan D. L., Werner C. (2016). ACS Biomater. Sci. Eng..

[cit28] Bray L. J., Binner M., Holzheu A., Friedrichs J., Freudenberg U., Hutmacher D. W., Werner C. (2015). Biomaterials.

[cit29] Xiao Y., Gao X., Taratula O., Treado S., Urbas A., Holbrook R. D., Cavicchi R. E., Avedisian C. T., Mitra S., Savla R. (2009). BMC Cancer.

[cit30] Wang Y., Yi S., Sun L., Huang Y., Lenaghan S. C., Zhang M. (2014). J. Biomed. Nanotechnol..

[cit31] Wagenblast E., Soto M., Gutiérrez-Ángel S., Hartl C. A., Gable A. L., Maceli A. R., Erard N., Williams A. M., Kim S. Y., Dickopf S., Harrell J. C., Smith A. D., Perou C. M., Wilkinson J. E., Hannon G. J., Knott S. R. V. (2015). Nature.

[cit32] Bersini S., Jeon J. S., Dubini G., Arrigoni C., Chung S., Charest J. L., Moretti M., Kamm R. D. (2014). Biomaterials.

[cit33] Seib F. P., Kaplan D. L. (2012). Biomaterials.

[cit34] Mu Q., Broughton D. L., Yan B. (2009). Nano Lett..

[cit35] Ky B., Putt M., Sawaya H., French B., Januzzi J. L., Sebag I. A., Plana J. C., Cohen V., Banchs J., Carver J. R. (2014). J. Am. Coll. Cardiol..

[cit36] Shi J., Kantoff P. W., Wooster R., Farokhzad O. C. (2017). Nat. Rev. Cancer.

[cit37] Gottesman M. M., Fojo T., Bates S. E. (2002). Nat. Rev. Cancer.

[cit38] Harris J., Klonoski S. C., Chiu B. (2017). Curr. Drug Delivery.

[cit39] Workman P., Aboagye E., Balkwill F., Balmain A., Bruder G., Chaplin D., Double J., Everitt J., Farningham D., Glennie M. (2010). Br. J. Cancer.

