# Supplementary material for: Soft and flexible poly(ethylene glycol) nanotubes for local drug delivery
Source: Nanoscale. 2018 May 1;10(18):8413–21. doi: 10.1039/c8nr00603b (PMC5944428; doi:10.1039/c8nr00603b)
Supplement: Supplementary file 1 [file NR-010-C8NR00603B-s001.pdf]

Electronic Supplementary Material (ESI) for Nanoscale.
This journal is © The Royal Society of Chemistry 2018

Soft and flexible poly(ethylene glycol) nanotubes for local drug delivery

Ben Newland, Christian Taplan, Dagmar Pette, Jens Friedrichs, Martin Steinhart, Wenxin Wang, Brigitte Voit, F. Philipp Seib, Carsten Werner*

Supporting Information

Experimental Section

All materials were purchased from Sigma and used as received unless otherwise stated.

Synthesis of Polymer Nanotubes: The general procedure for synthesizing the polymer nanotubes is adapted from previous work and shown in Scheme 1 of the main text.[1] Monomer stock solutions were prepared containing 5 % wt/vol of monomer dissolved in acetone together with 2-hydroxy-2-methylpropiophenone (HMPP) as a photoinitiator. For each monomer type used, the acrylate to photoinitiator molar ratio of 2.5:1 was used. Three diacrylate monomers were used in this study: poly(ethylene glycol) diacrylate (Mn = 575 g/mol)(for "PEG" nanotubes), bis[2-(methacryloyloxy) ethyl] phosphate (Mw = 322.25 g/mol) (for "Phos" nanotubes) and bisphenol A ethoxylate diacrylate (Mn = 688 g/mol) (for "Bisphenol" nanotubes). To create the nanotubes, 25 μL of the respective solutions were equally spread across both sides of a quarter of an anodized aluminum oxide (AAO) template (200 nm: AnodiscTM 47 with 200 nm pore size and 47 mm disc diameter, pore depth = 60 μm)[36-37] or 10 μL was spread onto the self-ordered AAO templates with 400 nm pores (pore depth either 10 or 100 μm, prepared based upon previous protocols[38-39]). The polymer infiltrated AAO was then flushed with nitrogen for two minutes and crosslinked by exposing it to UV light (DELOLUX 04 from DELO, UV wavelength = 315-500 nm, intensity: 78000 mW/cm^2) for 1 minute. To remove the template and yield free nanotubes, the AAO template was dissolved in 1 M sodium hydroxide solution. Three milliliters of sodium hydroxide were added per quarter of AAO template and left for 30 minutes with vortexing and sonication at the later stages to yield free nanotubes (optical observation). The obtained yellow-white dispersion was washed and centrifuged (13,000 rpm) twice with sodium hydroxide, three times with water and twice in ethanol, and subsequently dried over night at 40°C in a vacuum oven.

Polymer Nanotube Characterization: The nanotubes were characterized via light microscopy after the last purification step prior to drying them. Diluted nanotube samples in ethanol were dried in a 96-well plate and imaged using an Olympus IX73 light microscope (20x objective) in brightfield mode. Nanotube lengths were analyzed using FIJI image software, with 1800 nanotubes being measured per nanotube type by applying a macro kindly provided by Benoit Lombardot (MPI-CBG Dresden) and by measuring nanotubes manually where necessary. Scanning electron microscopy (SEM) was used for characterizing the AAO templates, assessing the overall form of the nanotubes and for measuring nanotube diameters. Samples for SEM were prepared by dispersing nanotubes in ethanol, drying the dispersion on a glass slide in a vacuum oven and transferring the nanotubes onto a SEM sample holder covered with a graphite based adhesive. AAO templates were simply pressed gently onto the graphite based adhesive. These samples were then sputtered with gold for 60 s at 40 mA (SCD 050 Sputter Coater, Balzers). SEM examination was performed on a XL30 ESEM-FEG (Philips) using the secondary electron detector and acceleration voltages of 3.0 - 5.0 kV. Transmission electron microscopy (TEM) was used for further analysis of the nanotubes using a Zeiss Libra 200 TEM with an accelerating voltage of 200 kV using the contrast aperture. Raman spectroscopy was performed via a Raman Imaging System alpha300R from WITEC investigating liquid PEGDA monomer and solid nanotube pellets. The settings used were: laser wavelength = 532 nm (5-10

mW); objective = 20x Zeiss, integration time = 0.5 s, accumulation = 200 - 500, wave number range = 3650 - 300 cm-1, including baseline correction, detector = CCD.
Nanotube Stiffness Characterization: Atomic force microscopy (AFM) measurements were performed using a Nanowizard IV AFM (JPK Instruments) mounted on an inverted optical microscope (Axio Observer A1, Zeiss). Measurements were conducted in MilliQ water at room temperature (25°C) using a Petri-Dish Heater (JPK Instruments) sample chamber. Immobilized polymer nanotubes were imaged in the QITM mode using a cantilever with a nominal spring constant of 0.3 N m-1 (qp-BioAC; Nanosensors). Spring constants were calibrated before measurements using routines implemented in the AFM acquisition software that are based on the Sader method.[40] The acquisition parameters employed were: 300 nm ramp, pixel time 10 ms and a force trigger of 1 nN. The data processing software provided by the AFM manufacturer (JPK Instruments) was used to extract the elastic modulus E from approach force-distance curves.

Cytotoxicity Analysis of Nanotube Suspensions: Light microscopy and the PrestoBlue assay were used to assess the cytotoxicity following incubation with a range of nanotube types and concentrations. Mouse fibroblasts (3T3) (NIH) were cultured in Dulbecco's Modified Eagle Medium (DMEM) (Gibco) supplemented with fetal bovine serum (FBS) (Biochrom) and 1% Penicillin/Streptomycin (P/S). Human mesenchymal stem cells (MSCs) were kindly provided by the laboratory of Professor Martin Bornhäuser which were isolated from male human bone marrow aspirates (donor age 20-40 years) (University Hospital Carl Gustav Carus, Technische Universität Dresden) as previously described.[2] MCF10A cells were cultured in DMEM/F12 with 15 mM HEPES buffer (Gibco) supplemented with 5% horse serum, 10 mg/mL insulin, 20 ng/mL epidermal growth factor (PeproTech), 100 ng/mL choleratoxine, 0.5 mg/mL hydrocortisone and 1% P/S. All cell culture work was carried out under standard sterile conditions, and cells were maintained in a humidified incubator with 5% CO_2 at 37°C. Cells were seeded at a density of 5,000 cells/well in 96-well plates 24 hours prior to experimentation. Polymer nanotubes (PEG, Bisphenol and Phos) or MWCNTs were dried in ethanol to a known mass of 240 μg. The nanotubes were then resuspended in the appropriate cell medium and dilution series from 120 to 7.5 μg/mL were prepared. The cell culture medium in the well plates was replaced with the nanotube dilution series, with control wells receiving just a change of medium. The well plates were then incubated for either one day or three days before analysis. Light microscopy was performed (Olympus IX73) then the PrestoBlue assay was carried out according to the manufacturer's protocol using media alone blanks and nanotube blanks. With the average blank value subtracted from the sample readings, the cell metabolic activity values were normalized to untreated control cells (i.e. termed 100% viable). Experiments were carried out in quadruplicate. Blank readings for all types of nanotubes, and doxorubicin, were performed to ensure that the nanotubes did not interfere with the assay.

Doxorubicin Loading and Release: Doxorubicin hydrochloride (LC Laboratories) was prepared in MilliQ grade water containing 0.1% vol/vol dimethyl sulfoxide (DMSO), by first dissolving the doxorubicin in the DMSO part then topping up with the appropriate volume of water. For assessing the loading capability of the four types of nanotubes, 0.5 mg of nanotubes was resuspended in 0.5 mL of 100 μg/mL doxorubicin solution. All loading experiments were carried out for 72 hours at room temperature. Doxorubicin solution (0.5 mL) was left for the incubation period to serve as the negative control (0% loading). The nanotubes were then centrifuged for 2 minutes at 13,000 rpm and the supernatant was assessed in comparison to the doxorubicin alone solution via absorbance measurement (DU 800 Spectrophotometer, Beckman Coulter, λ_{max} 481 nm). For determining the maximum loading capacity of PEG nanotubes the same protocol was performed but with various dry weights of nanotubes, each

being incubated with 200 μL of an 800 μg/mL doxorubicin solution and incubated for 24h at room temperature.

Release data was obtained using PEG nanotubes loaded at the concentrations that would be used for the *in vivo* study; 0.6 mg of PEG nanotubes were incubated for 72 hours with 100μL of either 400 μg/mL or 800 μg/mL doxorubicin solution. Next, nanotubes were pelleted by centrifugation, the supernatant was removed and replaced with phosphate buffered saline to form a 1 mg/mL nanotube in PBS suspension at 37°C. At each time point (from day 1 to day 42) the supernatant was collected and stored at 4°C in the dark and the pellet was resuspended in fresh PBS to continue the release study.

In Vitro Analysis of Doxorubicin Delivery to Breast Cancer Cell Lines: The breast cancer cell line MCF-7 (ATCC) was cultured in DMEM (4.5 g glucose, 110 mg sodium pyruvate) supplemented with 10% v/v FBS and 10 μg/mL insulin. The metastatic breast cancer cell line MDA-MB-231 (ATCC) was cultured in RPMI1640 medium supplemented with 10% FBS and 1% P/S. Cells were seeded at a density of 10,000 cells/well in a 96-well plate 24 hours prior to experimentation. PEG nanotubes and MWCNTs (240 μg) pre-loaded with 240 μL doxorubicin solution (80 μg/mL) were washed twice with sterile water then once with the appropriate cell culture medium (by centrifugation and removal of the supernatant) then diluted to final concentrations ranging from 120 μg/mL to 7.5 μg/mL. Unloaded nanotubes were diluted in a similar manner to act as a control. Doxorubicin solution diluted to 4 μg/mL in cell media was used as the positive control. The nanotubes were incubated on the cells for 1 day and 3 days, before light microscopy and PrestoBlue analysis was carried out as detailed above.

Focal Delivery of Doxorubicin to Othotopic Human Xenografts: All *in vivo* studies were approved by the Home Office of the United Kingdom (Project License Number PPL 70/8801). Animals were maintained under Home Office regulations and this study complied with best practice in cancer research.[3] Tumor xenografts were induced using MDA-MB-231-derived tumor cells that metastasize following orthotopic injection into mice; the tumors carried the firefly luciferase gene to permit *in vivo* bioluminescence imaging.[4] *In vivo* studies were based on protocols developed previously.[5] Briefly, female NOD/SCID mice (NOD.CB17-Prkdcscid/NcrCrl) aged 49 to 56 days were obtained from Charles River UK Limited. A total of 5×10^5 cells in 20 μL of Matrigel (BD Biosciences) were injected bilaterally into the 4th or 5th mammary fat pad. Following tumor induction, mice (group sizes 4 to 5) were treated at day 14. Mice received bilateral injections of 100 μL of PEG nanotubes loaded with either 40 μg doxorubicin ("standard" dose) or 80 μg doxorubicin ("high" dose) close to the tumor sites (i.e., total 80 μg of doxorubicin/mouse or 160 μg of doxorubicin/mouse) but were not given an intra-tumor injection. As a control, the equivalent doxorubicin dose of 80 μg was administered in 100 μL of normal saline via a bolus tail vein injection to the control mice. Disease progression was monitored weekly by tumor cell-associated bioluminescence imaging using the Xenogen IVIS 200 imaging system controlled by the Living Image Software 4.3.1 (Caliper Life Sciences). At the endpoint of the study, the brain, lung, liver and bones (hind legs) of each mouse were examined for metastasis using bioluminescence imaging, and the primary tumors were dissected and weighed.

Statistical Analyses: Data was analyzed using GraphPad Prism 6.07 (GraphPad) software. Nanotube stiffness was analyzed using a one-way ANOVA with Tukey's post hoc multiple comparisons test. Nanotube cytotoxicity was analyzed via a two-way ANOVA (treatment & dose) for each time point separately with a Tukey's post hoc multiple comparisons test. Tumor growth *in vivo* (bioluminescence) was analyzed using a two-way ANOVA (treatment &

time)(repeated measures by treatment) with Tukey's post hoc multiple comparisons test. Tumor weight was analyzed using a one-way ANOVA with Tukey's post hoc multiple comparisons test. Error bars represent the standard deviation throughout, except for the *in vivo* analysis, which represent the standard error of the mean. An asterisk denotes statistical significance as follows: $*P < 0.05$, $**P < 0.01$, $***P < 0.001$.

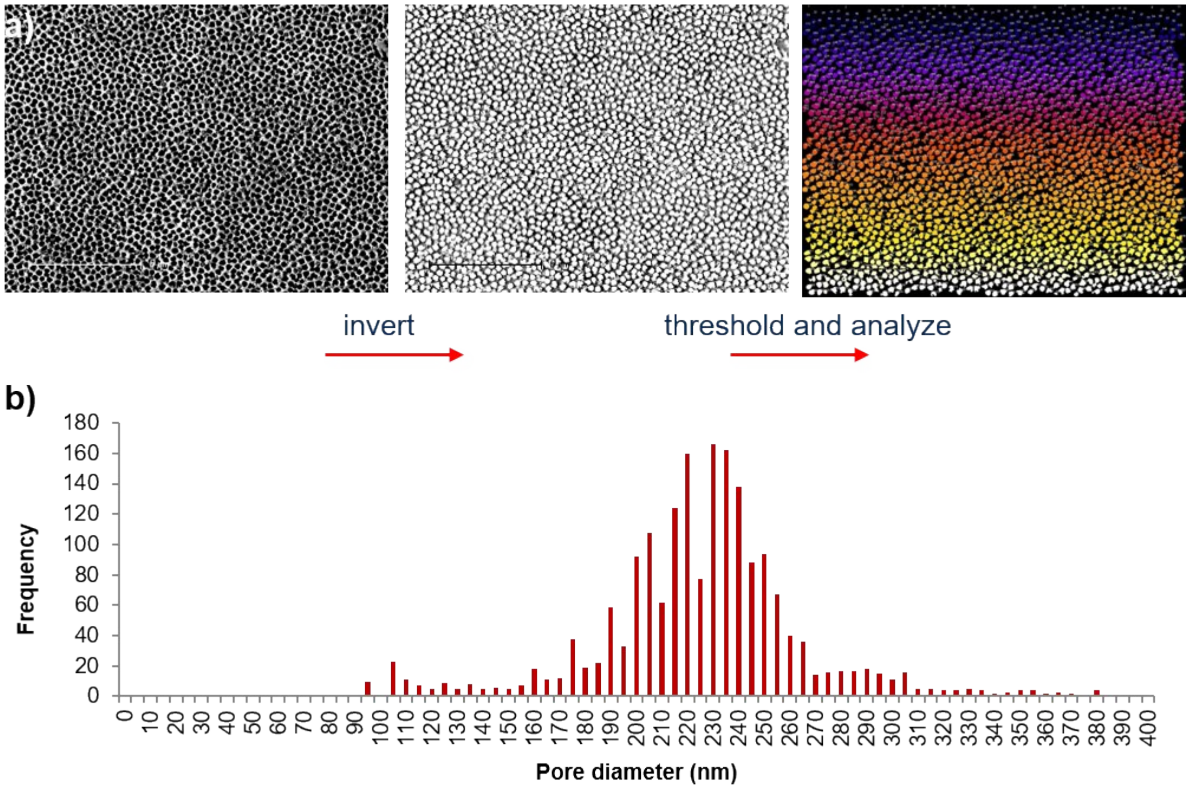

Figure S1 Anodized aluminum oxide (AAO) template characteristics. a) the image used and subsequent image processing to characterize the pore size of the Whatman AAO templates, and b) the resulting pore size distribution (1915 pores analyzed, average pore size 224 nm +/- 44nm). It should be noted here that this is only the distribution of the pore size of one surface of the template and does not necessarily represent the pore diameters at different depths throughout.

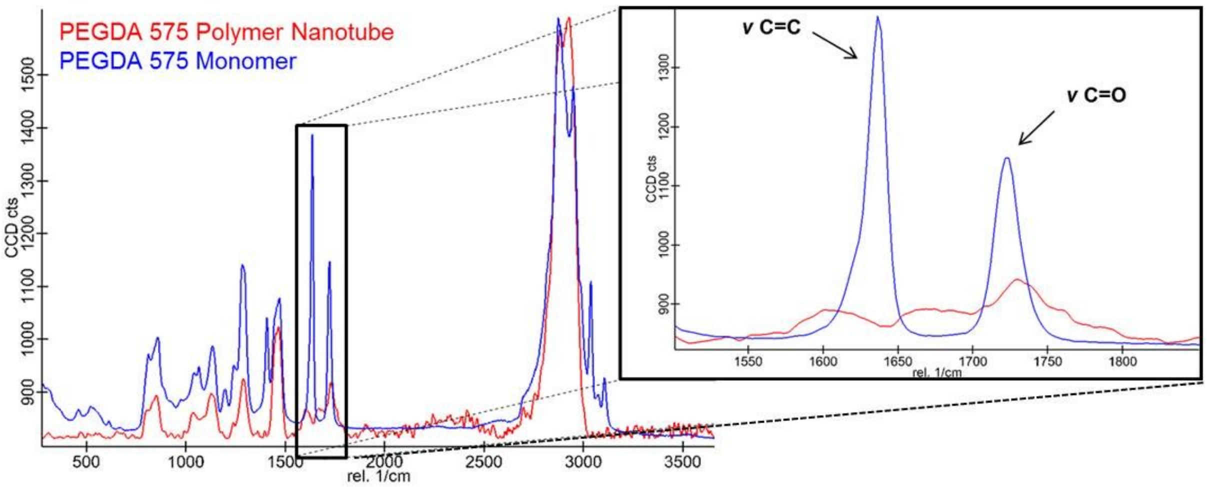

Figure S2 – Raman spectroscopy analysis of PEG nanotubes in comparison to the PEGDA monomer. Results show that the C=C signal of the acrylate group (v(C=C) at 1637 cm^{-1}, v(C=O) at 1730 cm^{-1}) is gone after the photopolymerization indicating complete crosslinking of the network.

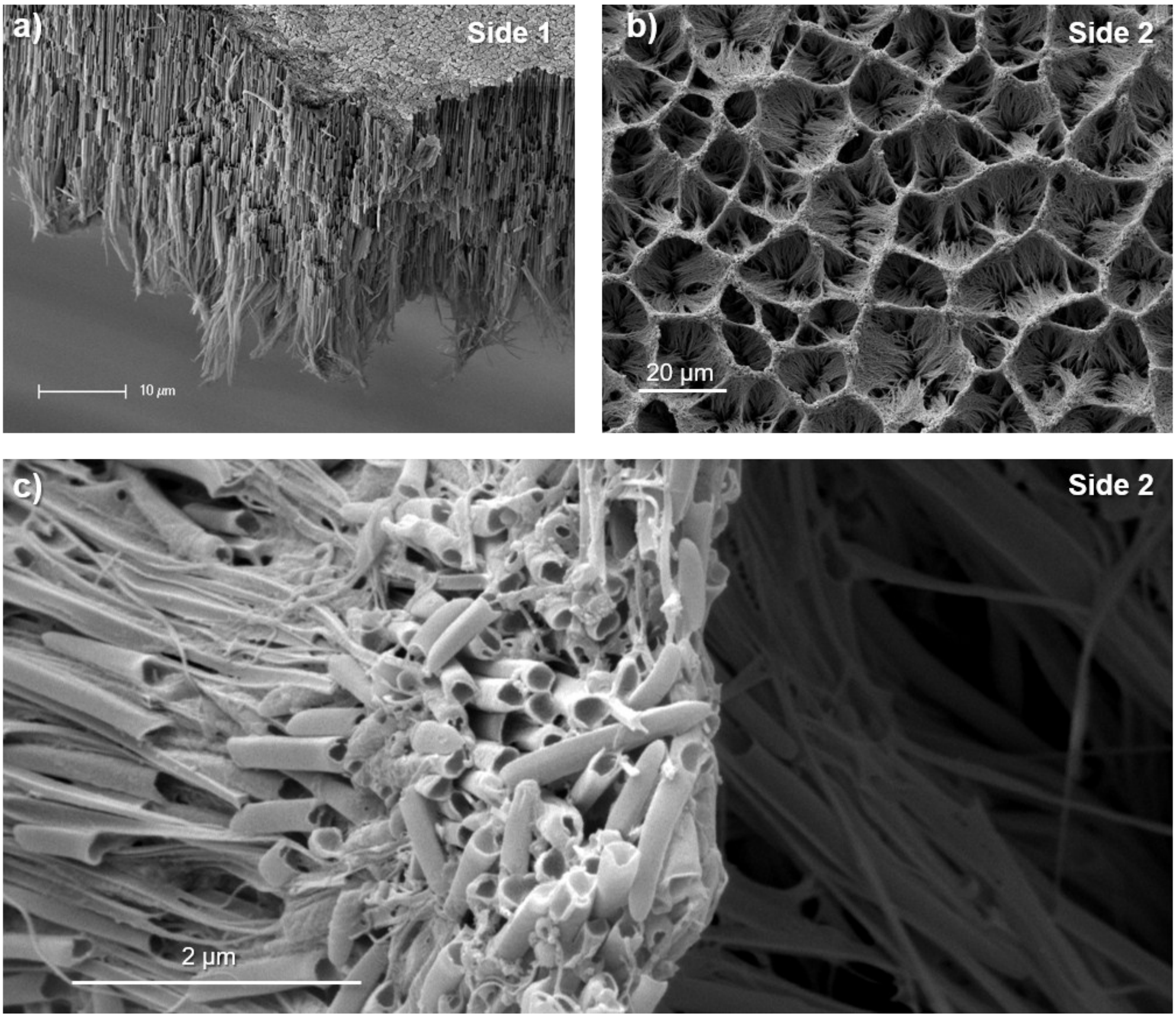

Figure S3 – Impact of sodium hydroxide treatment on anodized aluminum oxide templates. SEM images of the anodized aluminum oxide template, which is filled with polymer nanotubes, but has been subjected to 3 minutes treatment in sodium hydroxide to begin the dissolution process. a) Side 1 of the template which still looks largely intact compared to the other side (Side 2), b) which shows tube ends (further magnified in c) that are still held in place by the remains of the template.

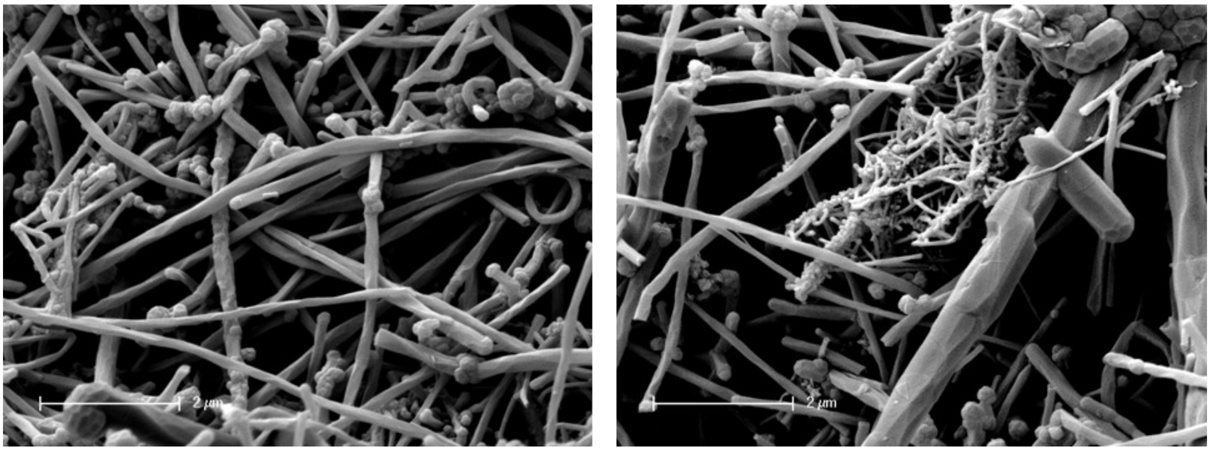

Figure S4 – SEM images of the MWCNTs shown in their pristine form as used throughout the studies. These images highlight the range of nanotube diameters (left) and the presence of non-tubular impurities (right).

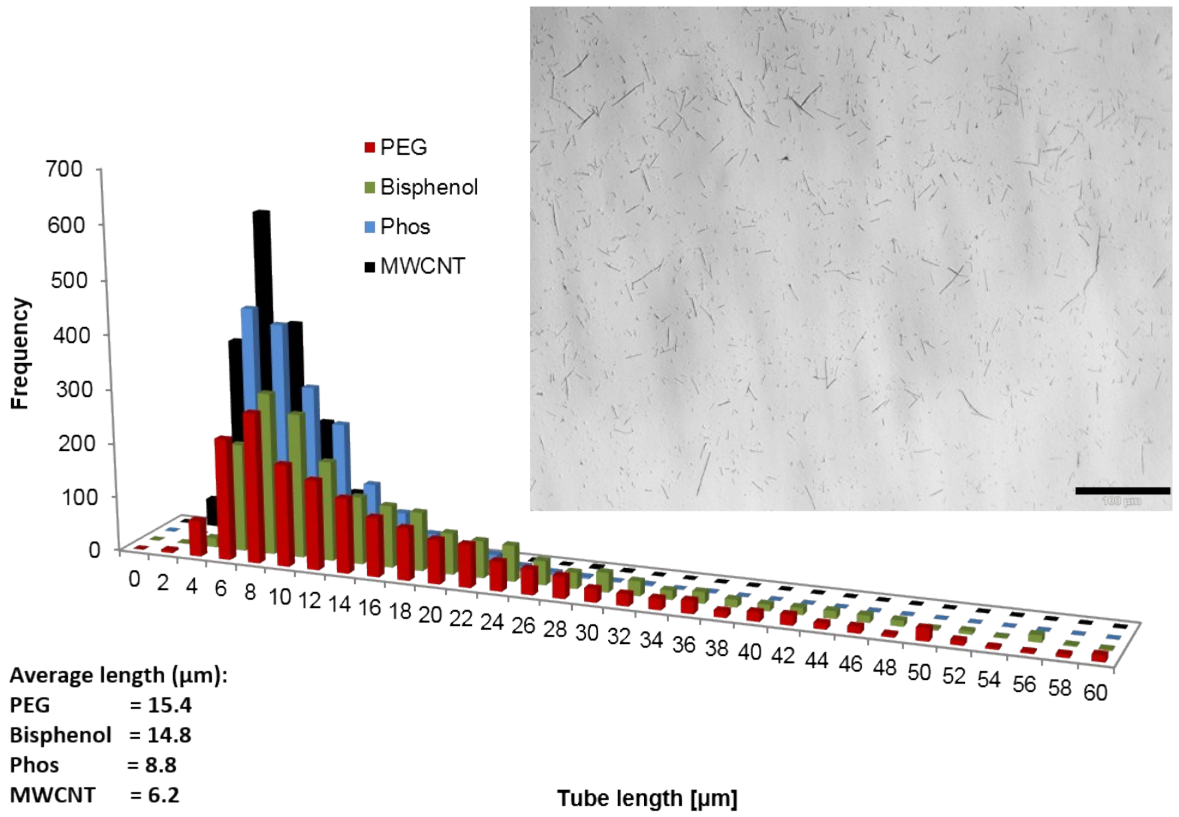

Figure S5 – Length comparison of polymer nanotubes. Nanotube length distributions of the three polymer nanotubes in comparison to multiwalled carbon nanotubes. An example light microscope image of PEG nanotubes is shown inset which is then used for length determination using a macro designed for FIJI software by Dr. Benoit Lombardot, Scientific Computing Facility, MPI-CBG, Dresden (scale bar = 100 µm).

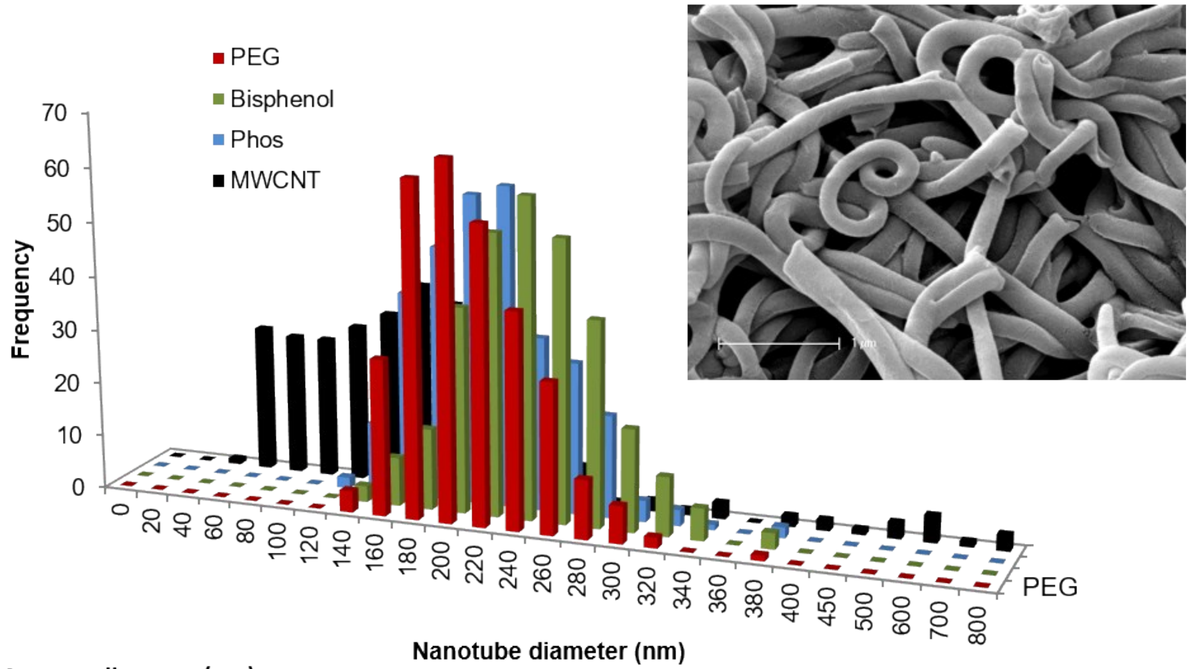

Average diameter (nm):
PEG = 202
Bisphenol = 233
Phos = 202
MWCNT = 164

Figure S6 – Characterization of the polymer nanotube diameters from SEM images (insert showing a typical SEM image of PEG nanotubes). MWCNTs show a much broader distribution of sizes compared to the three polymer nanotubes.

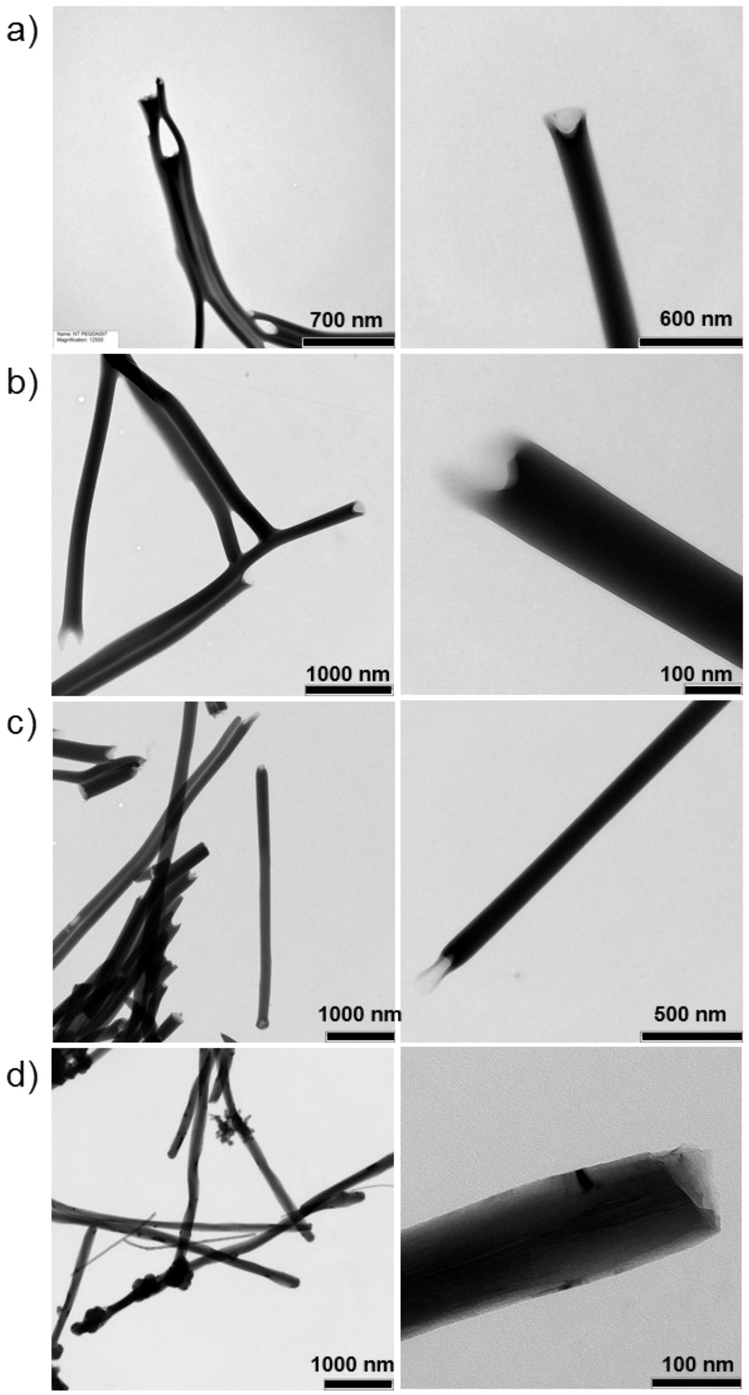

Figure S7 – Transmission Electron Microscopy (TEM) analysis of the different nanotube types. Low magnification (left hand side) and higher magnification (right hand side) of (a) PEG nanotubes, (b), Bisphenol nanotubes, (c) Phos nanotubes, (d) and MWCNT.

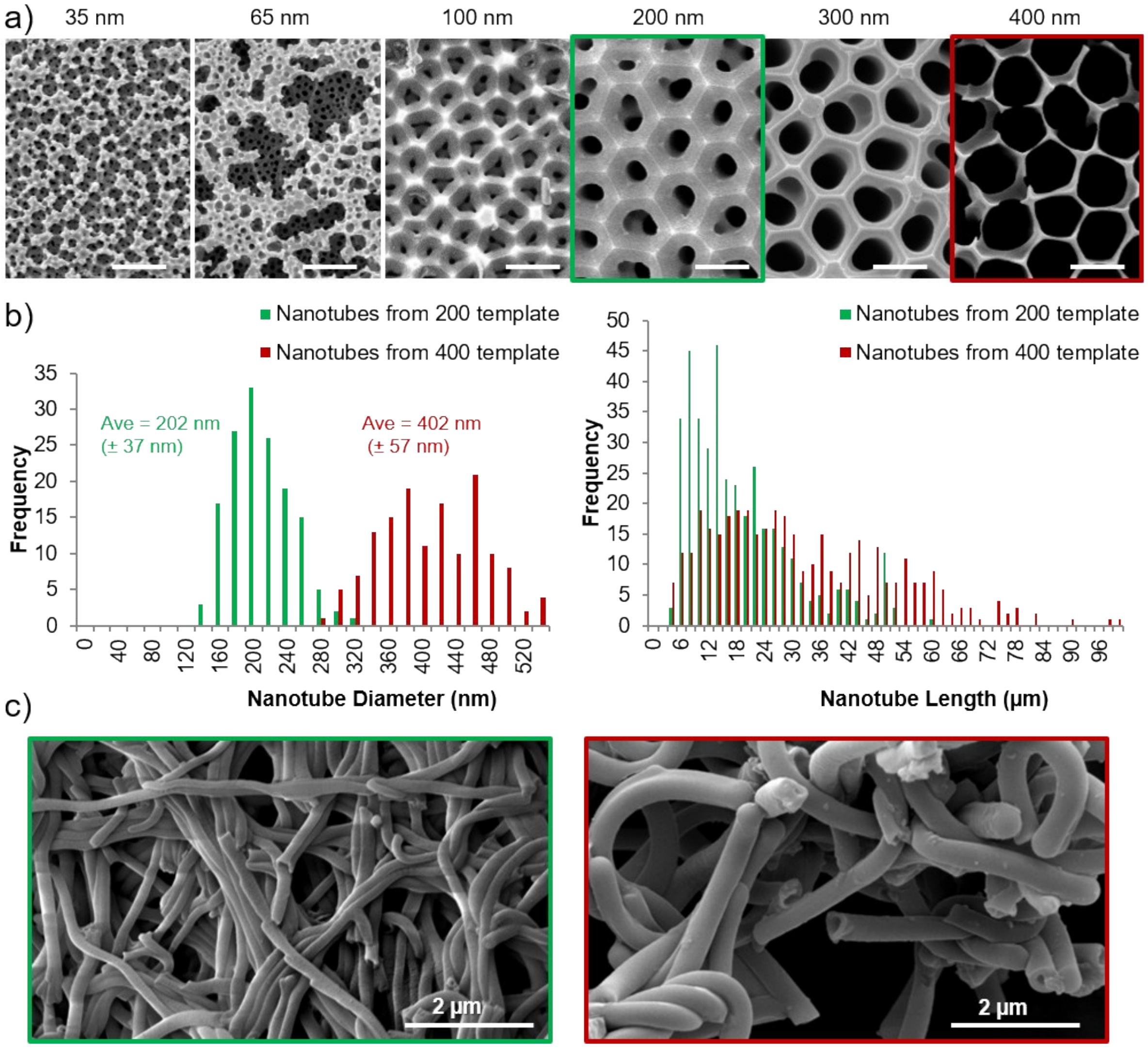

Figure S8 – Nanotube diameter properties are dependent on the template used. a) Scanning electron microsope (SEM) images of various anodized aluminum oxide templates (scale bar = 500 nm) with approximate pore size indicated above. b) Diameter and length distributions of PEG nanotubes synthesized in the 200 and 400 nm templates. c) SEM images of the resulting nanotubes showing the increase in diameter obtained when using the 400 nm template.

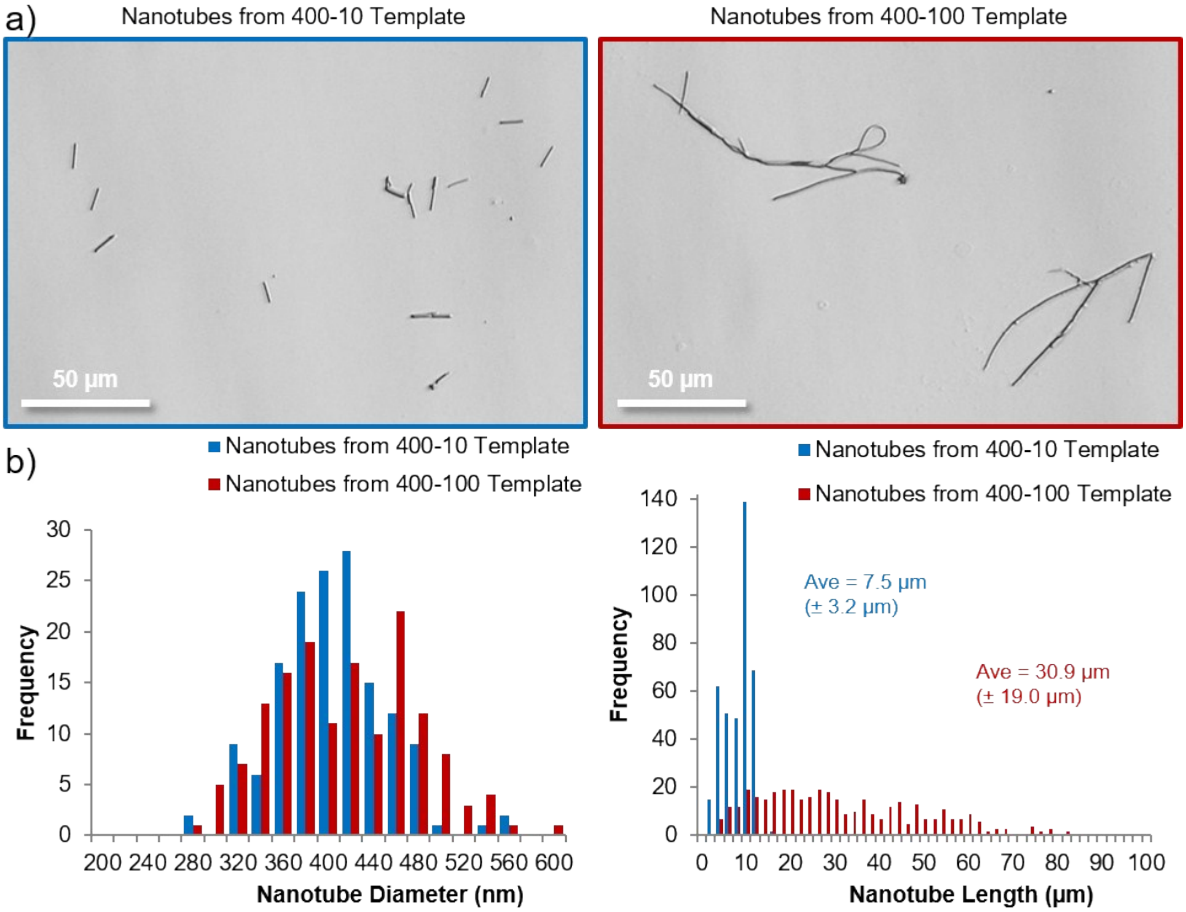

Figure S9 – Average nanotube length is dependent on the template used. a) Light microscope images of PEG nanotubes synthesized in the 10 µm deep template (400-10) and 100 µm deep template (400-100). b) Corresponding diameter and length distribution analysis shows that the nanotube lengths can be restricted to 10 µm whilst the diameter remains similar.

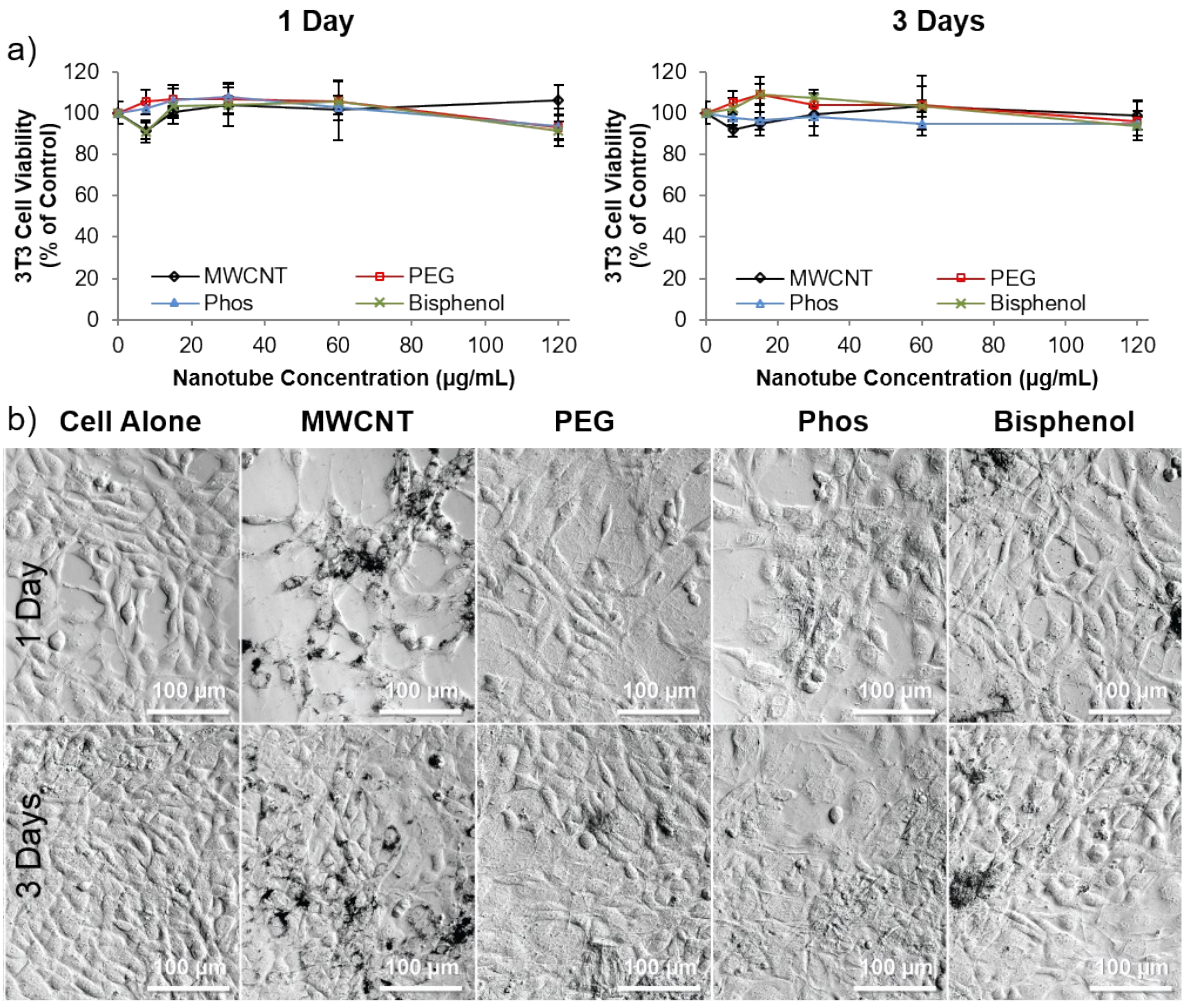

Figure S10 –Fibroblast cell viability after incubation with polymer nanotubes. a) The cell metabolic activity (measured using PrestoBlue) of 3T3 fibroblasts incubated with varying concentrations of nanotubes was normalized to cells receiving no treatment (marked as 100% viable). The PrestoBlue assay (which measures cell metabolic activity) was chosen for this study as we have previously shown that polymer nanotubes do not interfere with the assay (assessed again herein for each nanotube type (Figure S13)).Increasing the concentration of nanotubes up to 120 µg/mL did not result in a loss of viability after one day (left hand side) or three days of incubation (right hand side)(n=4 for each nanotube type, error bars represent ± standard deviation). b) Corresponding light microscope images of the 60 µg/mL nanotube concentration after 1 day (upper panel) or 3 days of incubation. Clusters of MWCNT can be observed on the cells with few nanotubes between the cells, whereas the PEG nanotubes form an even layer across the cells and well plate bottom.

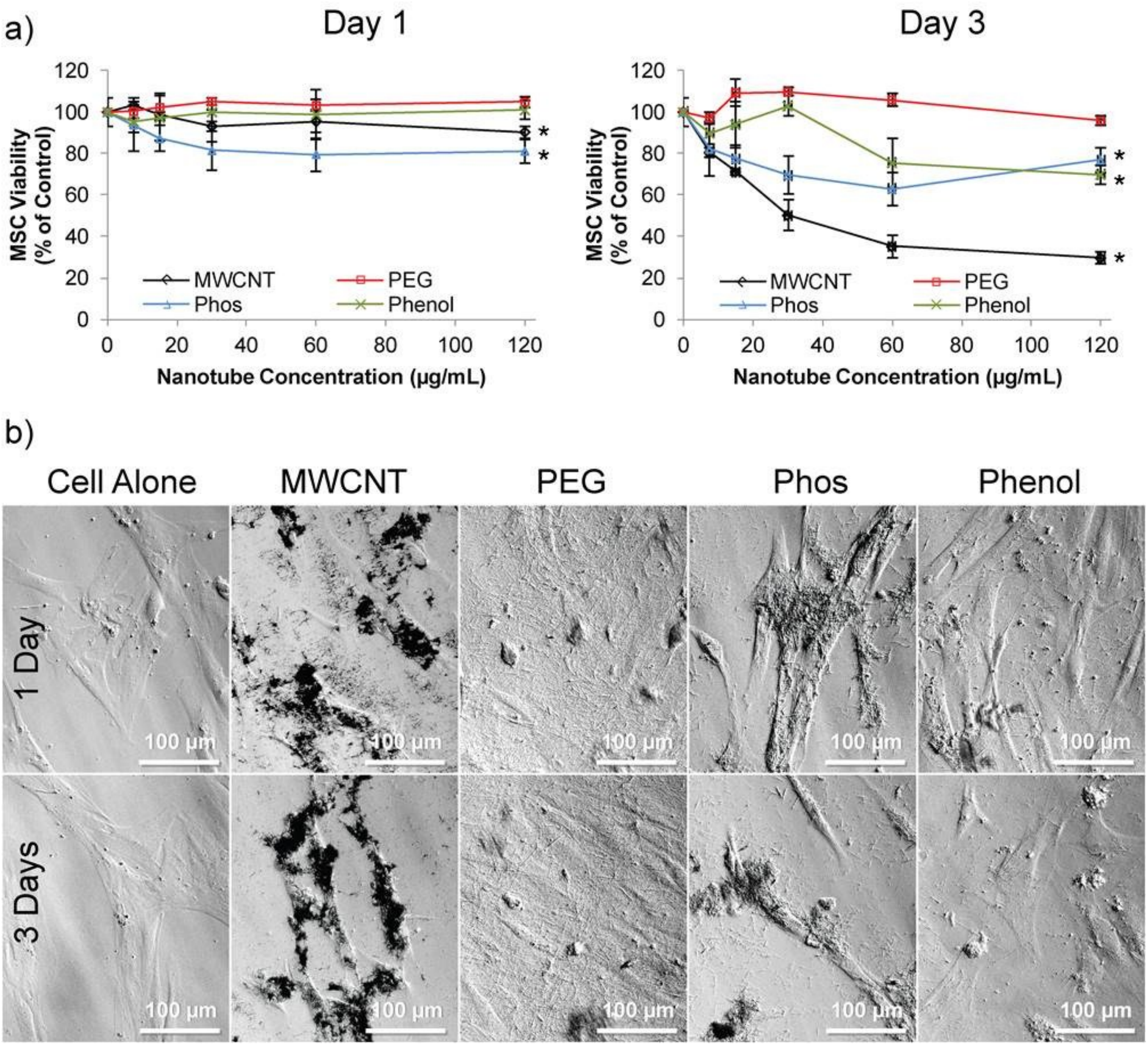

Figure S11 – MWCNTs reduce the viability of human mesenchymal stem cells (MSC). a) PrestoBlue analysis of MSCs after one day (left) or 3 days (right) of incubation with nanotubes at a range of concentrations. After three days the highest concentration of Phos nanotubes, Bisphenol nanotubes and MWCNTs reduced cellular viability significantly compared to PEG nanotubes (n=4, error bars represent ± standard deviation, * represents statistical significant ($P \leq 0.05$) reduction in viability compared to PEG nanotubes (asterisks only included at highest concentration for clarity)(two-way ANOVA with Tukey's multiple comparison test)) b) Corresponding light microscope images of the 60 µg/mL nanotube concentration show that both MWCNT and Phos nanotubes aggregate in vicinity of the cell whereas PEG and Bisphenol nanotubes form a more even layer across the cells and well plate bottom.

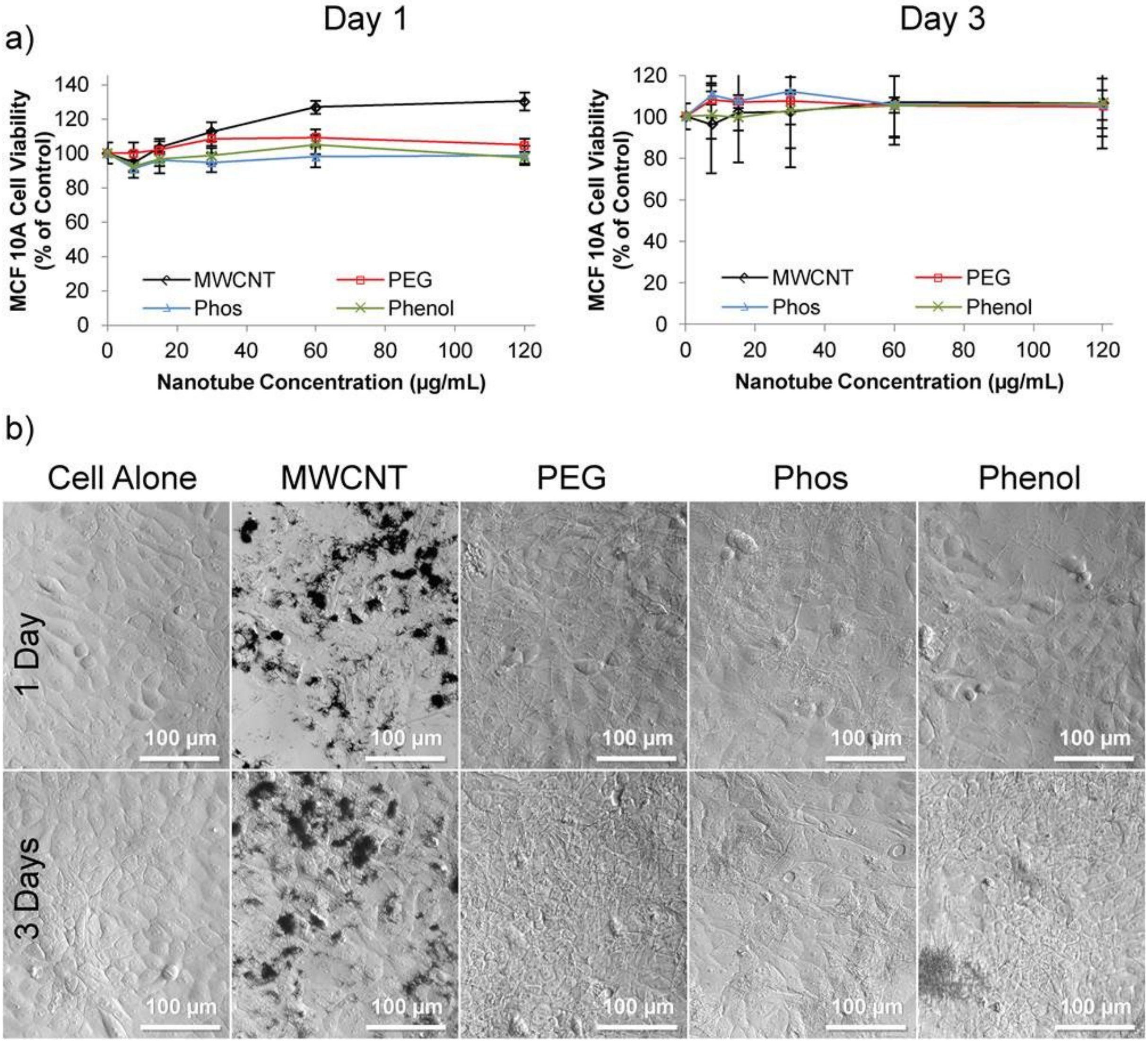

Figure S12 – Breast epithelial cells (MCF 10A) appear unaffected by incubation with nanotubes. a) PrestoBlue analysis of MCF 10A cells after one day (left) or 3 days (right) of incubation with nanotubes at a range of concentrations. No significant reduction in cellular viability compared to incubation with PEG nanotubes was observed (n=4, error bars represent ± standard deviation) b) Light microscope images of the 60 µg/mL nanotube concentration show aggregated MWCNTs

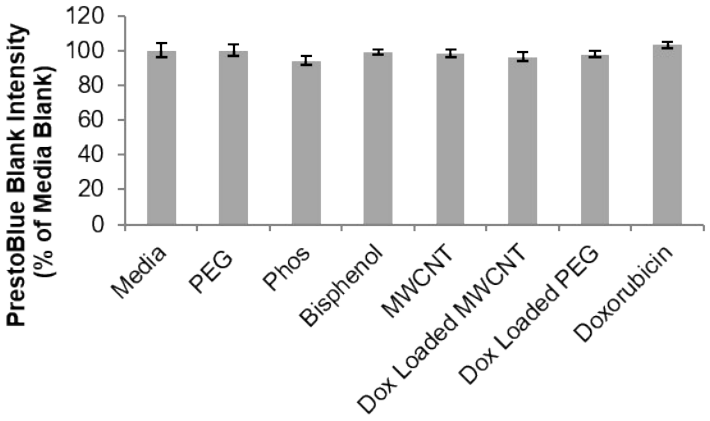

Figure S13 – PrestoBlue blank analysis to check that neither the nanotubes, doxorubicin, nor drug loaded nanotubes affect the assay. The assay was performed under the same conditions as all the in vitro toxicity analysis and incubated for the same period for PrestoBlue color change (30 minutes). Error bars represent ± standard deviation, n=3.

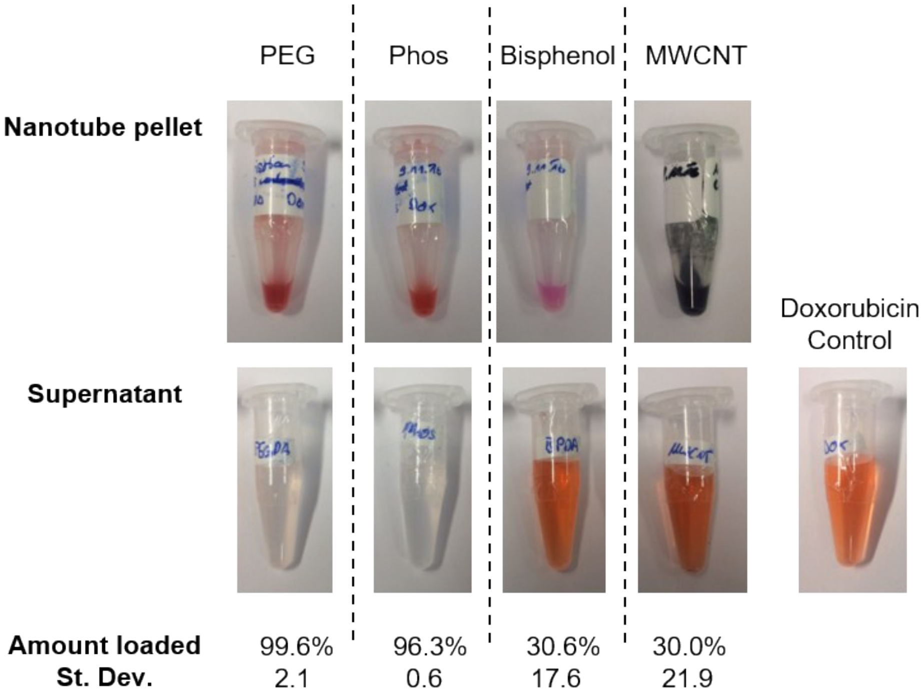

Figure S14 – Qualitative assessment of doxorubicin loading of synthesized nanotubes. PEG and Phos nanotubes show highest uptake of doxorubicin. 0.5 mg of nanotubes was loaded with 50 µg of doxorubicin for 24 hours. After this time the nanotubes were pelleted and the photographs of centrifuge tubes containing pellets (upper panel) or the supernatants (lower panel) were taken. The clear supernatants from the PEG and Phos nanotubes samples show visually that the doxorubicin was removed from solution to the nanotubes (see the dark red pellets in upper panel).

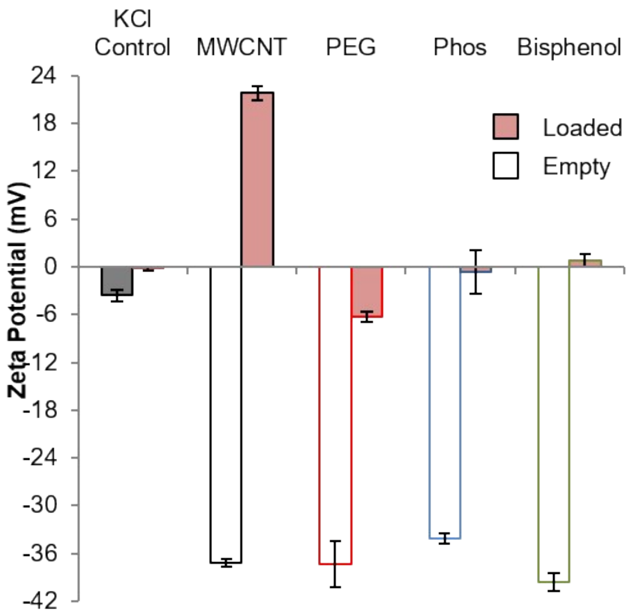

Figure S15 – Zeta potential analysis of the empty and loaded nanotubes. Empty nanotubes have a negative surface charge which becomes more positive when they are loaded with the positively charged doxorubicin molecule. (n = 3, error bars represent ± standard deviation).

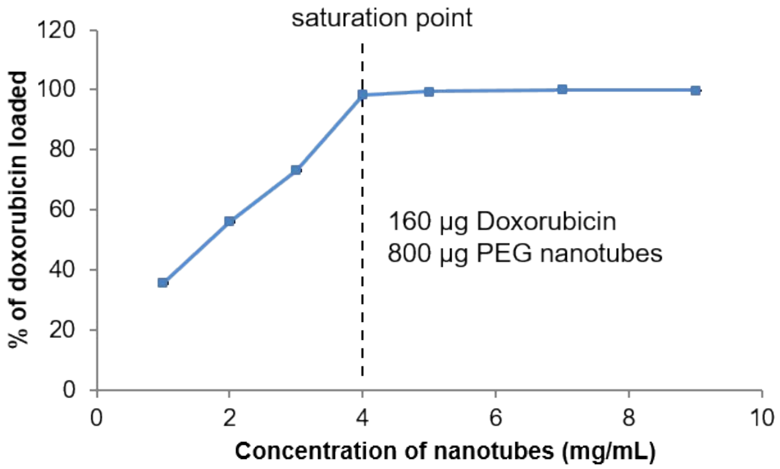

Figure S16 – Determination of the loading saturation point for PEG nanotubes. At PEG nanotube concentrations below 4 mg/mL not all the doxorubicin (160 µg) is loaded to the nanotubes. The weight to weight ratio of doxorubicin to PEG nanotubes was calculated (0.2 to 1) at the saturation point.

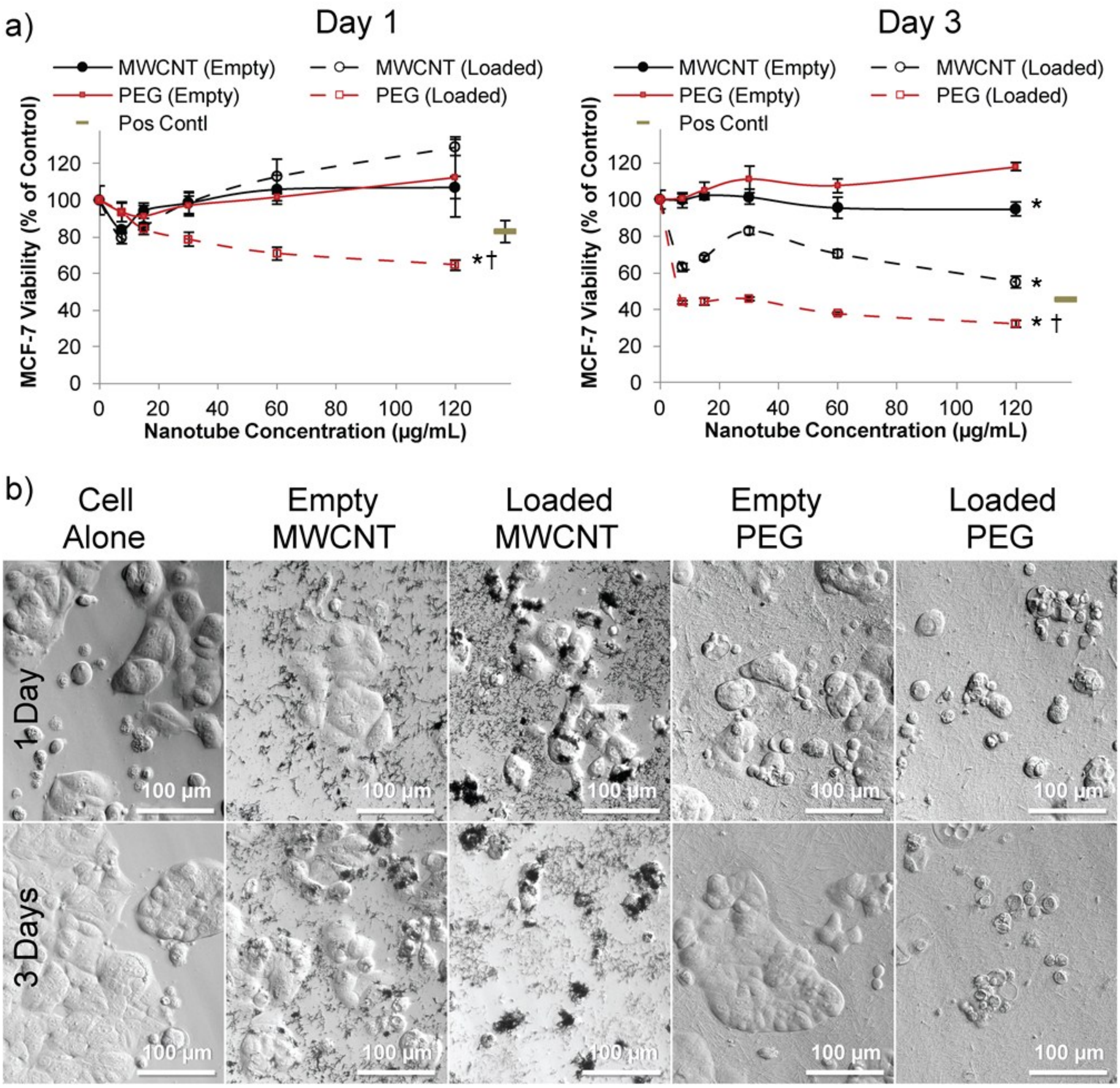

Figure S17 – Doxorubicin loaded nanotubes reduce the viability of human breast cancer cells *in vitro*. a) incubating MCF-7 cells with doxorubicin loaded PEG nanotubes for 1 day (left hand side) or 3 days (right hand side) reduces their viability compared to unloaded nanotubes as measured via the PrestoBlue assay. b) corresponding light microscope images of the 60 µg/mL nanotube concentration show the difference in morphology between cells without nanotubes or empty nanotubes, and cells incubated with doxorubicin loaded nanotubes. (n = 4, error bars represent ± standard deviation, * represents statistical significant difference to empty PEG nanotubes, † represents a difference between loaded MWCNT and loaded PEG nanotubes (two way ANOVA with Tukey's multiple comparison test, P≤ 0.05)). Positive control was 4 µg/mL free doxorubicin in solution.

1(a) B. Newland, L. Thomas, Y. Zheng, M. Steinhart, C. Werner and W. Wang, *Journal of Interdisciplinary Nanomedicine*, 2016, **1**, 9; (b) B. Newland, D. Leupelt, Y. Zheng, L. S. V. Thomas, C. Werner, M. Steinhart and W. Wang, *Scientific Reports*, 2015, **5**, 17478.

2 J. Oswald, S. Boxberger, B. Jørgensen, S. Feldmann, G. Ehninger, M. Bornhäuser and C. Werner, *Stem Cells*, 2004, **22**, 377.

3 P. Workman, E. Aboagye, F. Balkwill, A. Balmain, G. Bruder, D. Chaplin, J. Double, J. Everitt, D. Farningham and M. Glennie, *British Journal of Cancer*, 2010, **102**, 1555.
4 R. H. Goldstein, M. R. Reagan, K. Anderson, D. L. Kaplan and M. Rosenblatt, *Cancer Research*, 2010, **70**, 10044.
5 F. P. Seib and D. L. Kaplan, *Biomaterials*, 2012, **33**, 8442.
